# O22 An inflammatory journey from brain to skin: a case of En Coupe De Sabre scleroderma presenting with raised intracranial pressure, seizures, and uveitis

**DOI:** 10.1093/rap/rkaa054.010

**Published:** 2020-11-03

**Authors:** Chantelle Richards, Despina Eleftheriou, Kate Armon, Brinda Muthusamy, Peter Bale

**Affiliations:** r1 Addenbrooke's Hospital, Cambridge, United Kingdom; r2 Great Ormond Street Hospital, London, United Kingdom

## Abstract

**Case report - Introduction:**

Scleroderma is a rare connective tissue disease characterised by thickening and hardening of the skin resulting in increased collagen production. The predominant type of childhood scleroderma is localised scleroderma (LSc) which primarily involves the skin, fascia, muscle, and bone. En coup de sabre (ECDS) is a rare subtype affecting the frontoparietal region and is characterised by a unilateral indurated streak in the shape of a sabre sword wound.

ECDS is also associated with extracutaneous features which can manifest prior to the classical skin changes. We present the case of a 7-year-old boy with ECDS presenting with neurological and ocular findings.

**Case report - Case description:**

A seven-year-old boy presented with severe headache followed by a twenty min right sided focal seizure. He had a 3-month history of headaches associated with vomiting suggesting raised intracranial pressure and ophthalmology review revealed bilateral papilloedema. Viral studies, bacterial serology and autoimmune screen were negative. He had normal inflammatory markers. Brain MRI demonstrated left sided white matter changes, thought to be secondary to inflammation. The clinical impression was of atypical pseudotumor cerebri and Acetazolamide was commenced.

Intermittent headaches persisted, without vomiting but unilateral left uveitis was identified. This was treated with Dexamethasone eye drops and his Acetazolamide was switched to Topiramate. Further imaging excluded malignancy and MRA and MRV were negative for vasculitis and sinus thrombosis. Neuro-Behcet’s was considered in the presence of HLA-b51 positivity but he did not meet diagnostic criteria.

His parents reported a transient knee swelling and an intermittent red line on his forehead. The rest of systemic screen was negative. In the presence of raised pressure, inflammation on imaging and dexamethasone dependant uveitis, he was commenced on a weaning course of oral prednisolone.

On this treatment his papilloedema and uveitis resolved but his Topiramate dose was increased due to intermittent brief focal seizures. MRI changes remained stable. A year after first presentation, episodes of left sided facial flushing became prominent associated with an injected left eye and an asymmetrical reduction of subcutaneous depth developed. Infrared thermography revealed warmth on the left forehead in the Blaschkoid distribution. The diagnosis was revised to ECDS with neuroinflammation.

The patient was commenced on high dose steroids and Mycophenolate Mofetil (MMF). Seizures resolved, uveitis and papilloedema remain absent and his skin stabilised. Recently there has been concern regarding progression of a facial lesion on reducing to 5 mg of Prednisolone, and a further prednisolone course and optimised MMF were initiated.

**Case report - Discussion:**

ECDS is an unusual variant of linear scleroderma but causes significant cosmetic changes. The skin lesions can be hard to treat and the choice of medication depends on response and associated extracutaneous features.

The lesion is typically erythematous during the acute inflammatory phase, before exhibiting a sunken ivory appearance. It is usually unilateral, rare below the forehead but can extend into the hairline. Asymmetrical facial development can occur.

In a large multicentre study of 750 children with LSc the prevalence and the clinical features of extracutaneous findings were identified. In 92% of cases a skin lesion was the presenting feature of disease. 4% of cases had neurological involvement. This increased to 18% in patients with ECDS. The most frequent neurological symptoms were seizures and headaches. When neuroimaging abnormalities are identified they are primarily ipsilateral to the skin lesion.

Ocular involvement is reported and in another large multicentre study of children with LSc, the involvement of the anterior segment of the eye was the second most frequent condition. Anterior uveitis was the most relevant finding.

In most patients the development of neurological symptoms follows the recognition of cutaneous disease. In our case his headaches, seizures and uveitis were the presenting feature prior to development of the scleroderma lesion. The cutaneous features were initially intermittent and transient making diagnosis challenging and classical skin changes may have been slowed by the initial course of oral prednisolone for his neurological and ocular disease. Access to infrared thermography proved to be a helpful modality to confirm the inflammatory skin lesion and affirm the diagnosis.

**Case report - Key learning points:**

This case highlights to clinicians the importance of assessing for extracutaneous features of linear scleroderma of the face. The case further highlights that these findings, including focal seizures associated with non-specific unilateral inflammatory neurological lesions, raised intracranial pressure, and unilateral uveitis can be presenting features before the typical skin changes are visible. Intermittent erythema in a longitudinal linear distribution may be the first indication of cutaneous disease and repeated thorough cutaneous examination is vital.

Anterior uveitis can be completely unrelated to the site of LsC and is asymptomatic. Ophthalmic monitoring is recommended for all patients and is mandatory in those with lesions on the face and/or CNS involvement. ECDS should be considered in the differential for all patients presenting with unilateral anterior uveitis.

The cutaneous outcome for children with ECDS is variable and some lesions remain refractory to the most used disease modifying treatments including methotrexate and MMF. In some cases, the inflammation self terminates but loss of subcutaneous tissue and asymmetrical facial development remains. Fat transplant remains an option in for some cases. The lesions can be very disfiguring and can cause significant psychological distress and we recommend early referral to clinical psychology within the paediatric rheumatology team.

